# Merging of synchrotron serial crystallographic data by a genetic algorithm

**DOI:** 10.1107/S2059798316012079

**Published:** 2016-08-18

**Authors:** Ulrich Zander, Michele Cianci, Nicolas Foos, Catarina S. Silva, Luca Mazzei, Chloe Zubieta, Alejandro de Maria, Max H. Nanao

**Affiliations:** a Structural Biology Group, European Synchrotron Radiation Facility, 71 Avenue des Martyrs, 38000 Grenoble, France; b European Molecular Biology Laboratory, Hamburg Outstation, Building 25a, c/o DESY, Notkestrasse 85, 22607 Hamburg, Germany; c Laboratoire de Physiologie Cellulaire et Végétale, Universite Grenoble Alpes, CNRS, CEA, INRA, BIG, Grenoble, France; d Laboratory of Bioinorganic Chemistry, Department of Pharmacy and Biotechnology, University of Bologna, Bologna, Italy; e EMBL Grenoble, 71 Avenue des Martyrs, CS 90181, 38042 Grenoble CEDEX 9, France

**Keywords:** genetic algorithms, cluster analysis, serial crystallography

## Abstract

Recent advances in macromolecular crystallography have made it practical to rapidly collect hundreds of sub-data sets consisting of small oscillations of incomplete data. This approach, generally referred to as serial crystallography, has many uses, including an increased effective dose per data set, the collection of data from crystals without harvesting (*in situ* data collection) and studies of dynamic events such as catalytic reactions. However, selecting which data sets from this type of experiment should be merged can be challenging and new methods are required. Here, it is shown that a genetic algorithm can be used for this purpose, and five case studies are presented in which the merging statistics are significantly improved compared with conventional merging of all data.

## Abstract 2

A genetic algorithm is described and used to select which sub-data sets from a larger pool can be merged into a high-quality data set.

## 1. Introduction   

The merging of oscillation data from multiple crystals or from multiple positions on the same crystal can be an effective method for obtaining complete, high-quality data sets. The concept of merging data from multiple crystals is not new and indeed dates back to the early days of macromolecular crystallography. With the advent of microbeams, this concept was expanded to include multiple sub-data sets collected from a single crystal (Perrakis *et al.*, 1999 ▸). Nonetheless, the collection of a complete data set from a single crystal over a single oscillation range is still the dominant approach, largely owing to the difficulties in merging small sub-data sets, which is particularly apparent when merging data collected from multiple crystals. The inherent non-isomorphism between crystals is thought to often preclude the useful merging of data sets, thus limiting the use of serial data collection. Recently, however, there has been renewed interest in these kinds of experiments, spurred in no small part by the success of sample-delivery and analysis methods developed at free-electron lasers (FELs), most notably in the SFX (serial femtosecond crystallography) method (Boutet *et al.*, 2012 ▸; Chapman, 2015 ▸). Indeed, some FEL sample-delivery and data-analysis methods have recently been adapted and extended for use at synchrotron sources at both cryogenic and ambient temperatures (Gati *et al.*, 2014 ▸; Stellato *et al.*, 2014 ▸). One cryogenic method that takes advantage of the ability to collect small oscillation ranges from multiple crystals has recently been described by Zander *et al.* (2015 ▸). In this method, a diffractive map is first calculated, followed by the collection of ‘sub-data sets’ at the positions on the sample holder with the best diffraction properties. Because oscillation ranges, rather than still images, are collected using this method, the partiality of reflections can be determined more easily, reducing the number of images required to obtain a complete and high-quality data set. One problem that remains, however, is treating the non-isomorphism between crystals or between positions on a single crystal. This is highly dependent not only on the system being studied but also on the method of cryoprotection and other factors, including crystal nucleation and microenvironment growth conditions. In order to address these challenges, hierarchical cluster analysis has been the method of choice to select which data sets should be merged. This method uses some metric of similarity between data sets, most notably correlation coefficients between data sets, similarity of unit-cell parameters and relative anomalous correlation (Giordano *et al.*, 2012 ▸; Liu *et al.*, 2012 ▸; Foadi *et al.*, 2013 ▸). With the exception of anomalous correlation, these values are a proxy for the presumed data quality of the merged data, which is a severe limitation of this methodology. We therefore propose a simple method in which the data quality, as assessed by data metrics such as *R* values and 〈*I*/σ(*I*)〉, is directly optimized. However, for a set of *n* sub-data sets, the number of possible combinations is 2^*n*^ − 1; thus, an exhaustive search quickly becomes computationally unfeasible with even a small number of sub-data sets. In order to address this, we have therefore chosen to use global optimization as a means of identifying sets of sub-data sets that can be merged with good statistics. Genetic algorithms (GAs) are a well known global optimization method which have previously been used to address diverse problems in macromolecular crystallography (Chang & Lewis, 1994 ▸; Kissinger *et al.*, 1999 ▸; Schneider, 2002 ▸; Uervirojnangkoorn *et al.*, 2013 ▸). Here, we show that a GA can be used to select which sub-data sets can be merged into a high-quality data set and present test cases as proof of concept.

## 2. Materials and methods   

### 2.1. Sample preparation   

#### 2.1.1. Glucose isomerase   

A slurry of glucose isomerase crystals was purchased from Hampton Research. Crystals were cryoprotected by dilution of the 5:1 protein:100% glycerol slurry to a final concentration of 20% glycerol.

#### 2.1.2. Ultralente insulin   

Microcrystalline ultralente insulin was provided by Gerd Schluckebier (Novo Nordisk) and David Flot (ESRF). The crystal slurry was cryoprotected in the same manner as glucose isomerase.

#### 2.1.3. Thermolysin   

Thermolysin from *Bacillus thermoproteolyticus* (Sigma–Aldrich) was dissolved in 45% DMSO, 0.05 *M* MES pH 6.0 at a concentration of 100 mg ml^−1^. Crystals were grown using the hanging-drop vapour-diffusion method, where the drops were composed of the protein solution and a solution consisting of 0.05 *M* MES pH 6.0, 1 *M* NaCl, 45% DMSO in a 1:2 ratio. The reservoir contained 35% ammonium sulfate. Crystals were cryoprotected by transferring them for 5 s into a drop containing 6 *M* trimethylamine *N*-oxide.

#### 2.1.4. LUX–DNA complex   

The LUX–DNA complex (the DNA-binding protein LUX ARRHYTHMO from *Arabidopsis thaliana* in complex with its cognate DNA) was expressed, purified and crystallized as described in Silva *et al.* (2016 ▸). LUX–DNA crystals were cryoprotected by adding approximately 1/10 volume of precipitant solution to the crystallization drop, followed by harvesting.

#### 2.1.5. Urease   

*Sporosarcina pasteurii* urease (SPU) was purified following a previously reported protocol (Mazzei *et al.*, 2016 ▸). Subsequently, 2 µl urease solution was diluted with 2 µl precipitant solution (1.6–2.0 *M* ammonium sulfate in 50 m*M* sodium citrate buffer pH 6.3). Crystallization was performed at 293 K using the hanging-drop method, equilibrating the drop against 0.5 ml precipitant solution using Qiagen EasyXtal 15-well plates. Rice-shaped protein crystals appeared in 1–2 weeks and grew to dimensions of 20 × 20 × 40−70 µm. Crystals were transferred into a cryoprotectant solution consisting of 20% ethylene glycol and 2.4 *M* ammonium sulfate in 50 m*M* sodium citrate buffer pH 6.3.

### 2.2. Data collection   

Following cryoprotection, crystals were harvested in either nylon loops (glucose isomerase, ultralente insulin) or Kapton meshes (LUX-DNA, thermolysin, urease; Mitegen, USA) and flash-cooled in a gaseous nitrogen stream at 100 K. A diffractive map was first obtained using the *MeshAndCollect* workflow (Zander *et al.*, 2015 ▸) running within *MXCuBE* (Gabadinho *et al.*, 2010 ▸). This diffractive map was used as described by Zander and coworkers to determine the data-collection parameters for a series of sub-data sets (Table 1 ▸). No attempt was made to control the orientation of crystals in sample holders, nor was any selection of data sets performed based on their orientation.

Data for urease crystals were collected on the EMBL P13 beamline at the PETRA III storage ring, c/o DESY, Hamburg, Germany (Cianci *et al.*, 2016 ▸) equipped with an Arinax MD2 running on a 240 Hz CPU for fast grid scanning and a Dectris Pilatus2 6M. Data for LUX-DNA, glucose isomerase and ultralente insulin were collected on ESRF beamline ID23-EH2 equipped with an MD2M and a Dectris Pilatus3 2M (Flot *et al.*, 2010 ▸). Data for thermolysin were collected on ESRF beamline ID29 equipped with a microdiffractomer and a Dectris Pilatus2 6M (de Sanctis *et al.*, 2012 ▸). Doses were estimated using *RADDOSE*-3*D* (Zeldin *et al.*, 2013 ▸).

### 2.3. Integration   

Data were automatically integrated by *XDS* (Kabsch, 2010 ▸) running within the *GreNADES* automatic processing system (Monaco *et al.*, 2013 ▸). Where applicable, data sets were re-indexed for consistency across all sub-data sets using the REFERENCE_DATA_SET keyword in *XDS*.

### 2.4. Urease phasing   

Experimental phasing was performed with *SHELXC*, *SHELXD* and *SHELXE* (Sheldrick, 2010 ▸). A partially refined model was obtained by molecular replacement in *Phaser* (McCoy *et al.*, 2007 ▸) using PDB entry 4ceu (Benini *et al.*, 2014 ▸), followed by refinement in *phenix.refine* (Adams *et al.*, 2010 ▸). Known S-atom positions were obtained using this model and *ANODE* (Thorn & Sheldrick, 2011 ▸). Phase errors against this structure were computed in *SHELXE*.

### 2.5. Hierarchical cluster analysis (HCA)   

The merging of sub-data sets based on hierarchical cluster analysis was performed as described by Giordano *et al.* (2012 ▸) using a new GUI (Santoni *et al.*, unpublished work). This program reads the output of *XDS* (XDS_ASCII.HKL), calculates the correlation coefficients between each pair of data sets and saves them as a distance matrix. From this matrix the clustering dendrogram is generated and presented in an interactive GUI. By the selection of different nodes in this dendrogram, different combinations of data sets are generated and automatically processed in the background with *XSCALE*. The final correlation coefficient cutoffs are specified in Table 2 ▸.

### 2.6. Paired refinement   

Paired refinement was performed as described previously (Karplus & Diederichs, 2012 ▸; Diederichs & Karplus, 2013 ▸) except that resolution increments were selected to keep the numbers of reflections similar. Refinement of atomic positions and individual atomic displacement parameters was performed using *phenix.refine*, with simulated annealing in the first round of refinement. Evaluation of *R* values (without refinement) was performed using *phenix.model_vs_data* and the ‘high_resolution’ keyword (Adams *et al.*, 2010 ▸). The same free reflections were used to calculate *R*_free_ at high and ‘low’ resolution. The starting PDB entries used were 4zb5 (Lobley *et al.*, 2016 ▸), 3tt8 with copper(II) removed (B. Prugovecki & D. Matkovic-Calogovic, unpublished work) and 5a3y (Zander *et al.*, 2015 ▸) for glucose isomerase, insulin and thermolysin, respectively. The final optimized resolutions can differ from the resolution limits used for GA optimization and reported in Table 2 ▸.

### 2.7. Genetic algorithm   

**Figure 1:**
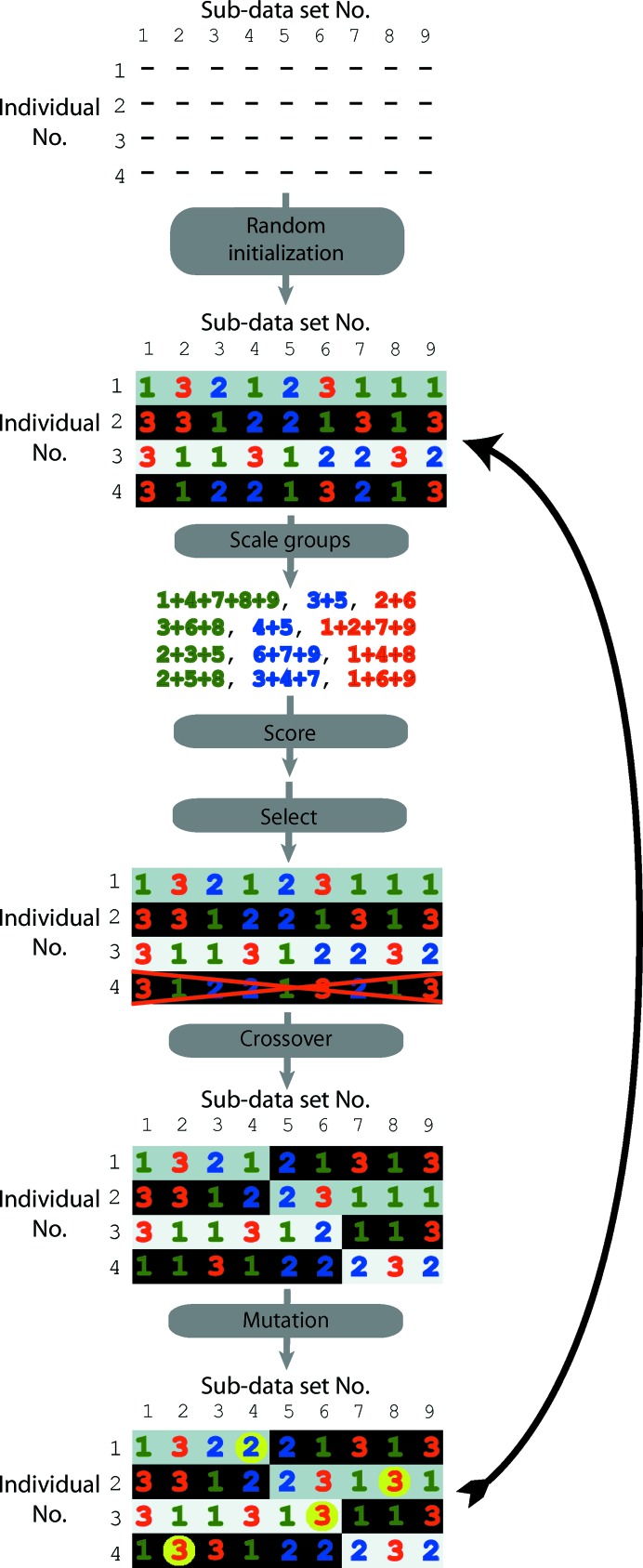
Schematic diagram of the genetic algorithm steps. In this example there are four individuals, with nine sub-data sets to be segregated into three groups. The individuals are first initialized randomly; the nine sub-data sets are assigned randomly to group 1, 2 or 3. Within an individual, three scaling runs in *XSCALE* are then performed, one for each group. The merging statistics are then converted to fitness scores, and the individual receives the fitness for the highest group (it is also possible to use the average fitness). In this case, individual 4 is removed from the population because of lower fitness (fitness values are not shown) and replaced with a new individual. The *DEAP* built-in mutation and crossover genetic modifiers are then applied, followed by cycling back to the scoring step. The background colour indicates the source of the chromosome. For example, after the crossover step between individuals 1 and 2, two ‘new’ individuals are created consisting of (i) the group assignments of sub-data sets 1–4 from individual 1 and the group assignments of sub-data sets 5–9 from individual 2 and (ii) the group assignments of sub-data sets 5–9 from individual 1 and the group assignments of sub-data sets 1–4 from individual 2. After crossover, mutations are randomly introduced as shown (yellow circles).

GAs apply concepts of biological natural selection to maximize or minimize a target function. The problem being optimized is encoded into one or more chromosomes, which are contained in a population of randomly initialized individuals. Diversity is introduced into the population *via* random mutation and crossover events. As a proof of concept, a GA for the grouping of sets of data sets has been implemented in a Python script. The *DEAP* package (https://github.com/deap/deap) offers a complete set of tools for the facile development of a GA and has therefore been used. Furthermore, the *SCOOP* package (https://github.com/soravux/scoop/) has been used for thread-level and host-level parallelization. In our implementation, a chromosome is an array of integers of length *n*, where *n* is the number of sub-data sets (Fig. 1 ▸). Each integer specifies which merging group each sub-data set belongs to. Thus, the range of each integer is limited to 1 … *g*, where *g* is the number of possible merging groups (three groups and *g* = 3 by default). A chromosome therefore simply describes how all of the sub-data sets should be divided into groups. Note that this encoding of data-set grouping implies no overlap between merging groups (one sub-data set cannot belong to more than one group). The algorithm proceeds as follows: a population of individuals, each containing a single chromosome, is first randomly initialized (Fig. 1 ▸) and then undergoes cycles of GA optimization by repeated selection, crossover between individuals, mutations and evaluations of fitness. The *DEAP* *EASimple* pre-built algorithm was used for this purpose, using uniform crossover (*p* = 0.05), uniform mutation (*p* = 0.05) and tournament selection (tournament size = 3) methods. Crossover and mutation probabilities can be user-specified and default to 0.3 and 0.6, respectively. The evaluation of individuals is performed by first scaling together all of the sub-data sets in the chromosome with the same group number (*g*) with *XSCALE* (Kabsch, 2010 ▸). Because the statistics are highly dependent on the binning of the data, resolution limits are user-selectable, either by specifying the maximum resolution or directly supplying a list of resolution shells which will be passed on to *XSCALE* *via* the RESOLUTION_SHELLS keyword. By default, the binning is automatically determined by *XSCALE*. After *XSCALE* has been executed, data-quality statistics are parsed from the XSCALE.LP file and a fitness is calculated, which is derived from the merging statistics. This fitness is a combination of the inner-shell *R*_meas_ value, the inner-shell 〈*I*/σ(*I*)〉, the outer-shell CC_1/2_, the overall completeness and the overall multiplicity. In cases where anomalous signal is present, a term for the anomalous signal can also be included in the scoring function, which is the addition of the inner-shell mean anomalous differences in standard deviations above the mean (SigAno in *XSCALE*/*XDS*). A second option for anomalous optimization exists, which is the resolution at which the SigAno remains above 1.0 and the anomalous correlation (‘% of correlation between random half sets of anomalous intensity differences’; *XSCALE* output) remains above 30%. The *DEAP* pre-built *EASimple* algorithm has been configured to maximize the fitness function. All components of the scoring function are therefore consistent with this (*i.e.* higher values are better) except for the second anomalous method and *R*_meas_. The *R*_meas_ term is therefore modified to be 100 − (*R*_meas_) (default) or 1/*R*_meas_. Each individual term also has a user-specified weight associated with it. All terms are then summed to produce a single score for each group in the individual. These statistics can be calculated for the inner resolution shell, the outer resolution shell or all of the data. Because the low-resolution bins contain the strongest data and are less influenced by the uneven distribution of multiplicity than the overall data (Karplus & Diederichs, 2015 ▸), we have chosen to use this resolution shell, except in the cases of multiplicity, completeness and CC_1/2_. Specifically, multiplicity and completeness use the overall statistics and CC_1/2_ uses the outer-shell statistics. Once each group in an individual has been scored, there are two options for how these group scores are converted into a fitness for the individual: the score from the best group or a combination of all of the group scores is used as the fitness of the individual. In the case where there is a single major dominant species, the two options should produce identical results. However, in cases in which there are multiple non-isomorphic groups, and the goal is to segregate these groups, scoring an individual by combining the scores across all groups is most appropriate. In this study, none of the test cases showed evidence of having several non-isomorphic groups, so we have focused on scoring from the best group.

### 2.8. Parameterization   

As with other optimization algorithms, finding an appropriate balance between weighting terms can in principle be problematic. In our GA implementation, there are two categories of parameters, all of which are available from the command line: algorithmic parameters such as population size, number of generations and crossover/mutation probabilities and parameters related to the scoring such as *R* weight, completeness weight and CC_1/2_ weight. In practice, we have found that the default GA parameters generally produce excellent results. However, if specific metrics appear to be sub-optimal, the GA formulation makes it straightforward to improve other metrics by simply changing the respective weights, lending additional versatility to the method.

## 3. Results and discussion   

### 3.1. Glucose isomerase   

**Figure 2:**
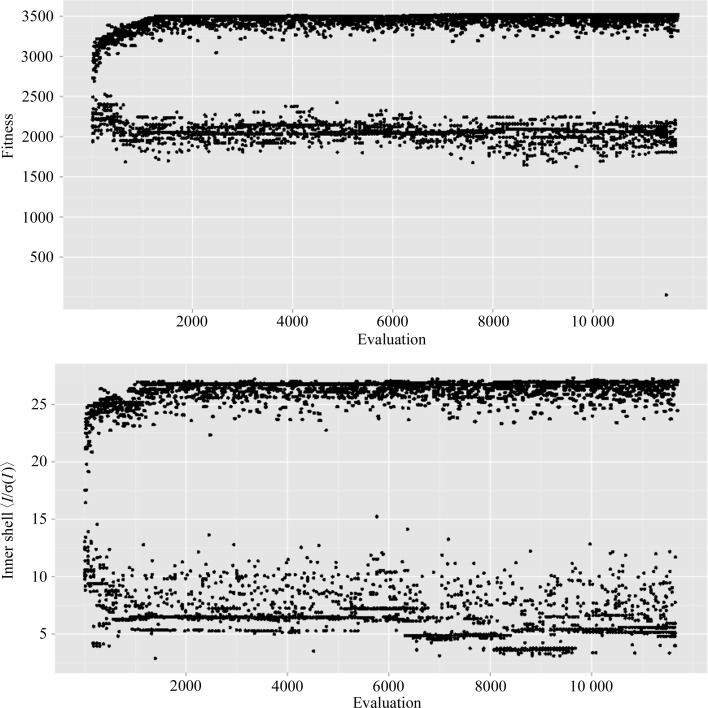
Improvement of data statistics. The horizontal axis represents progress of the algorithm. Upper panel: GA fitness is improved by the algorithm. Lower panel: the inner-shell 〈*I*/σ(*I*)〉 segregates into individuals with optimized values and suboptimal values.

Glucose isomerase crystals were used for initial testing of the GA. A small set of data sets (30 sub-data sets) were collected and merged (Tables 1 ▸ and 2 ▸). This yielded an acceptable merging *R*_meas,inner_ of 15.2% and 〈*I*/σ(*I*)〉_inner_ of 27.9. However, the overall *R*_meas_ of 35.1% was quite high. We were initially concerned that the strong correlation between various metrics could cause instability or nonconvergence of the GA, but found that this was not the case: submitting the sub-data sets to the GA for optimization showed a rapid improvement and convergence of the best fitness, with concomitant improvement of merging statistics (Fig. 2 ▸). Indeed, *R*_meas,overall_ was improved to 21.4%. Similarly, *R*_meas,inner_ was improved from 15.2% for all data to 9.9%. Finally, the CC_1/2_ values for the overall data set (CC_1/2,outer_ = 68.0%, CC_1/2,overall_ = 99.2%) were also improved using the GA (CC_1/2,outer_ = 70.8%, CC_1/2,overall_ = 99.5%). The 〈*I*/σ(*I*)〉 values, however, were not improved. Removing individual terms from the fitness function did not strongly affect the convergence rate of the GA. Thus, the GA appears to be effective in improving various metrics of data quality. Since the current standard for selective merging of sub-data sets is hierarchical cluster analysis (HCA), we also compared the overall data and GA-derived data with HCA data. This analysis showed that, as with GA, the 〈*I*/σ(*I*)〉 values were not significantly improved, but significant improvements to the *R*_meas_ could be made. Similar improvements of the CC_1/2_ to those with the GA could be made with HCA, although the outer CC_1/2_ value was slightly lower for HCA than for both all merged data and GA-optimized merged data. Although exploring the relationship between merging statistics and model quality was not the goal of this study, and indeed has been well studied by Karplus & Diederichs (2012 ▸), we nevertheless evaluated the downstream effects of these different merging methods using the paired refinement protocol. In this method, the high-resolution cutoff is incremented to include higher resolution data, followed by conventional refinement and finally evaluation of the resultant model against lower resolution data (Karplus & Diederichs, 2012 ▸). We found that the highest resolution shells in which the overall free *R* value decreased when evaluated against the previous resolution cutoffs were 1.87, 1.90 and 2.26 Å for the GA, HCA and all data sets, respectively. This suggests that the merging statistics are indeed indicative of improvements to the model quality.

### 3.2. Ultralente insulin   

Microcrystalline ultralente insulin is an excellent test system for serial crystallography and microcrystallography because of its stability, ease of cryoprotection and high-resolution diffraction. It is also useful for testing the global optimization approach because the crystals generally do not merge well together. 53 sub-data sets were collected from ultralente insulin crystals in a nylon loop (Tables 1 ▸ and 2 ▸). The *R*_meas_ from merging all data is particularly poor, with inner-shell and overall values of 44.8 and 35.3%, respectively. HCA identified a set of 19 sub-data sets from this pool with a significantly better *R*_meas_ values of 7.6 and 10.2% for the inner shell and overall, respectively. The 〈*I*/σ(*I*)〉 for the inner shell and overall were, however, lower than those on merging all data. The GA also selected a set of sub-data sets with considerably improved *R*_meas_ and signal to noise. The *R*_meas_ for the GA demonstrated an improvement over both merging all data and the HCA set, with an inner-shell value of 7.2% and an overall value of 9.7%. In contrast to the HCA set, the GA retained an inner-shell and overall 〈*I*/σ(*I*)〉 of comparable strength to merging all the data, and indeed showed somewhat higher values in the low-resolution bin. The CC_1/2_ values for merging all data were already very good, with CC_1/2,outer_ and CC_1/2,overall_ values of 77.4 and 99.3%, respectively. This was actually better in the outer shell than the HCA set (77.4% *versus* 67.3%). The GA produced CC_1/2,outer_ and CC_1/2,overall_ values that were better in both the outer and overall shells compared with merging all data and with the HCA (CC_1/2,outer_ = 79.1% and CC_1/2,overall_ = 99.8%). Despite the significant improvements to *R*_meas,inner_, paired refinement saw very modest differences in the high-resolution cutoff: 1.48, 1.52 and 1.52 Å for the GA, HCA and overall data, respectively. This result is consistent with the smaller improvements seen in CC_1/2_ and 〈*I*/σ(*I*)〉, lending further credence to the idea that these latter metrics are more useful than *R* values, as suggested previously (Karplus & Diederichs, 2015 ▸).

### 3.3. Thermolysin   

To test a case in which strong non-isomorphism was present, we collected data from three different sets of thermolysin crystals to give a total of 206 sub-data sets (Tables 1 ▸ and 2 ▸). These data surprisingly yielded a quite strong 〈*I*/σ(*I*)〉 for the overall and the inner resolution shells (15.3 and 66.2, respectively). *R*_meas_, however, was extremely poor for both the inner shell and overall (39.2 and 91.6%, respectively). HCA produced a data set with a significantly improved *R*_meas_ (*R*_meas,inner_ = 12.0%, *R*_meas,overall_ = 25.5%), but possibly because of reduced multiplicity the 〈*I*/σ(*I*)〉 was significantly worse in both the inner shell and overall [〈*I*/σ(*I*)〉_inner_ = 27.3 and 〈*I*/σ(*I*)〉_overall_ = 12.9]. Using default values, the GA initially produced a data set composed of 66 sub-data sets with extremely high 〈*I*/σ(*I*)〉 values [〈*I*/σ(*I*)〉_inner_ = 192.8 and 〈*I*/σ(*I*)〉_overall_ = 20.1]. The *R*_meas_, while an improvement over that on merging all the data, was rather high (*R*_meas,inner_ = 25.4%, *R*_meas,overall_ = 98.3%). By increasing the number of groups to eight from the default of three, and down-weighting the multiplicity term by 3, the merging statistics were dramatically improved, with *R*_meas,inner_ = 9.4%, 〈*I*/σ(*I*)〉_inner_ = 99.4 and 〈*I*/σ(*I*)〉_overall_ = 17.0. The 〈*I*/σ(*I*)〉 was dramatically higher for GA *versus* HCA, while the overall *R*_meas_ was slightly higher than for the HCA data set, but was still a significant improvement over that on merging all data. The CC_1/2_ values, in contrast, were somewhat lower than for both HCA and overall data, although increasing the weight for the CC_1/2_ term could produce a data set with an overall CC_1/2_ of 98.8% and an even higher 〈*I*/σ(*I*)〉 [〈*I*/σ(*I*)〉_inner_ = 211.94 and 〈*I*/σ(*I*)〉_overall_ = 31.79], but at the expense of a higher *R*_meas_ (*R*_meas,inner_ = 14.1%, *R*_meas,overall_ = 65.6%). This data set shows that in cases of high non-isomorphism it can be helpful to increase the number of merging groups and/or sacrifice multiplicity in order to improve 〈*I*/σ(*I*)〉 and *R*_meas_. Paired refinement revealed that the resolution of the GA data and HCA were both higher than the overall data, with values of 1.60, 1.65 and 1.76 Å, respectively.

### 3.4. LUX–DNA complex   

Lack of completeness can be of particular concern in SX experiments, owing to the fact that many sample-delivery techniques can favour specific orientations of crystals. One of the best-known examples of this is the alignment of rod-shaped crystals in a liquid jet. This problem is exacerbated in low-symmetry space groups, which require greater angular ranges for complete data sets. We therefore performed a *MeshAndCollect* SX experiment on the DNA-binding domain (AT3G46640.1, residues 139–200) of the LUX protein in complex with DNA, which crystallizes in space group *P*1 (Tables 1 ▸ and 2 ▸). 204 sub-data sets were collected, which when merged produced a complete and high-multiplicity data set. However, the merging statistics were extremely poor, with an *R*_meas_ of 72.7% in the low-resolution shell and of 76.1% overall as well as an 〈*I*/σ(*I*)〉 of 11.8 in the low-resolution shell and 7.8 overall. We wondered whether enough completeness and/or multiplicity could be sacrificed in order to improve both the *R*_meas_ and signal to noise, and whether there was a selection that could yield acceptable values for all parameters. The GA selected a set of data with significantly improved merging statistics. *R*_meas,inner_ was improved to 18.8%, 〈*I*/σ(*I*)〉_inner_ was increased to 16.3, 〈*I*/σ(*I*)〉_overall_ was improved to 9.9 from 7.8 and the overall completeness was still quite acceptable at 98.0% (compared with 99.9% for all data). As with the GA, HCA also improved the *R*_meas_ values compared with the overall data set, but not as significantly as with the GA, with *R*_meas,inner_ = 35.1%. While the GA improved the CC_1/2_ of the inner shell and that for the outer shell was worse than for the overall data, the HCA data set had an improved CC_1/2_ for both the inner shell and overall data. However, the 〈*I*/σ(*I*)〉 in the inner and overall shells actually decreased slightly compared with the overall data, with 〈*I*/σ(*I*)〉_inner_ = 11.3 and 〈*I*/σ(*I*)〉_overall_ = 7.5. Thus, the GA can produce high-quality results even in low-symmetry systems. A fully refined model was not available for paired refinement of this system, but the partially refined model (*R*_work_ = 0.26, *R*_free_ = 0.36, r.m.s.d. bond lengths = 0.011 Å, r.m.s.d. angles = 1.261°) was used to obtain resolutions of 2.76, 2.87 and 3.01 Å for GA, HCA and overall, respectively.

### 3.5. Urease   

A set of sub-data sets were collected at low energy from crystals of urease (Tables 1 ▸ and 2 ▸). The goal of this experiment was *de novo* phasing using endogenous S atoms and bound Ni^2+^ ions (there are 31 S atoms and two Ni^2+^ ions in the asymmetric unit and 799 amino acids). To this end, the anomalous signal was also included in the GA scoring function. Merging all data yielded very good 〈*I*/σ(*I*)〉 values overall and in the inner and outer resolution shells (24.9, 101.1 and 3.4, respectively) as well as excellent CC_1/2_ values of CC_1/2,outer_ = 80.5% and CC_1/2,overall_ = 99.9%. The mean anomalous differences divided by their standard deviation (SigAno in *XSCALE*) indicated the presence of anomalous signal with a low-resolution bin value of 3.16. Despite these generally favourable metrics, *R*_meas,inner_ was extremely poor (93.6%), as was *R*_meas,overall_ (86.2%). It is perhaps not surprising that phasing was unsuccessful using these data. In challenging SAD cases, it is frequently the substructure-determination step that prevents successful phasing of the data. We therefore tested whether it would be possible to determine interpretable phases starting from the known correct S-atom substructure. This was not possible, with a best weighted mean phase error (wMPE) of 83° and a CC of the partial model in *SHELXE* of 9.5%. We therefore applied the GA to these data. The best data set from the GA had CC_1/2_ values that were slightly lower than those for the overall data (CC_1/2,outer_ = 66.6% and CC_1/2,overall_ = 99.8%). However, the inner-shell 〈*I*/σ(*I*)〉 was significantly higher from the GA (121.4), while the overall value was slightly lower (23.3 *versus* 24.9). The SigAno was significantly higher than the overall data, with a SigAno_inner_ of 3.79. Finally, the *R*_meas_ values were dramatically improved (*R*_meas,inner_ = 6.7% and *R*_meas,overall_ = 30.1%). This set of data, despite having considerably better merging statistics, was still not of adequate quality for *de novo* phasing in *SHELX* (Sheldrick, 2010 ▸), *AUTOSHARP* (Vonrhein *et al.*, 2007 ▸), *CRANK*2 (Pannu *et al.*, 2011 ▸) or *PHENIX* (Bunkóczi *et al.*, 2015 ▸). However, as with the overall data, we were interested in whether the merged data were of adequate quality to produce interpretable phases starting with the known sulfur substructure. In this case, phasing was successful, with 590 of 799 amino acids automatically built, a wMPE of 34° and a CC of the partial model of 34.2%. Interestingly, despite the significantly better *R*_meas,inner_ of the GA data compared with the HCA merged data of 6.7 and 8.4%, respectively, as well as a larger SigAno_inner_ (3.79 *versus* 3.27), phasing from the known structure was similarly successful with the HCA.

## 4. Summary and future outlook   

In recent years, considerable effort has gone into the analysis of which merging statistics are linked to model quality and phasing success (Karplus & Diederichs, 2012 ▸, 2015 ▸; Diederichs & Karplus, 2013 ▸; Diederichs, 2016 ▸). Although some metrics such as CC_1/2_ and CC* appear to be much more generally useful than, for example, the classic merging *R* value, the specific combination of metrics that one uses is likely to be dependent on the downstream application. Here, we have shown that a GA can be used to select subsets of data that have improved merging statistics compared with merging all data. Indeed, in all of the test cases studied, significant improvements to the GA-derived statistics compared with merging all data have been observed. This can be performed automatically with minimal user intervention and is therefore suitable for inclusion in automatic pipelines. We feel that as the popularity of methods, including SX, that produce hundreds of sub-data sets increases, such an automatic tool will be extremely useful, especially in the not uncommon case where there is non-isomorphism between crystals. It is worth noting that other global optimization algorithms such as simulated-annealing and Monte Carlo methods could also be effective in this goal.

We have focused on obtaining a single high-quality data set in this work. In other words, optimization of the best single group is the goal. However, the encoding of chromosomes in our GA approach also supports the somewhat different aim of identifying multiple mutually non-isomorphous groups. This can be addressed by making the fitness proportional to, for example, the average fitness across all groups for a particular individual. In cases of multiple non-isomorphous groups, where the non-isomorphism can be distinguished *via* merging statistics, this would likely be a more appropriate approach. However, small changes such as slightly different ligand-binding modes or changes in loop conformations are unlikely to be distinguishable by such an approach. The serial data-collection approach offers the opportunity for a systematic analysis of the limits of these changes.

While we have compared the results of the GA with HCA analysis, the two approaches can be combined. For example, HCA can be used as an initial pre-selection, followed by GA optimization. This would take advantage of the sensitivity of HCA to outliers and could be performed in a fully automatic manner, setting a very strict similarity threshold in the HCA. Normally, the threshold used for HCA may require optimization by inspection of a cluster dendrogram, and this manual intervention step would be obviated if the initial threshold for HCA were set to a very high value. This approach would take advantage of the speed of the HCA and the ability of the GA to identify combinations of data sets that are not apparent based on CC or unit-cell parameters (for example if the number of reflections in sub-data sets is very low). Similarly, in cases where the sub-data sets have an adequate number of reflections, pre-screening can be performed based on the statistics within these sub-data sets. It is, however, worth noting that for all of the cases discussed here such an approach on its own (*i.e.* without GA) produced inferior data sets to HCA and GA.

The completeness of the sub-data sets in our study varied from very low (∼5% for LUX) to quite high (46% for thermolysin). Indeed, for LUX the number of reflections present in each sub-data set was so low that no meaningful merging statistics were produced by *XDS*/*XSCALE*. While high-quality data sets could still be obtained in this challenging case, it is likely that with even smaller wedges scaling could become impossible using standard methods, and techniques similar to those used for merging single images from serial femtosecond crystallographic experiments might become necessary.

As with HCA, the GA has parameters that can be changed, such as mutation and crossover probabilities. These can, in principle, affect the success and convergence rate. In practice, changing these values has rarely been necessary. Crystallo­graphic parameters are also parameterized, but we view this as an advantage, since one can directly select which metric or metrics are the most important. This is in most cases more intuitive than setting a correlation coefficient cutoff, a unit-cell similarity cutoff or a linkage method since the quantitative relationship between these parameters and the merging statistics, while directly related, is less obvious.

Several improvements to the GA implementation are envisioned, including the use of a faster (but possibly less robust) step for the determination of merging statistics. Although mitigated by the parallelization within *XSCALE* as well as the host-level and thread-level parallelization in the GA, because of the typically large number of sub-data sets and reflections *XSCALE* is currently the rate-limiting step. Run times are typically on the order of an hour on a 12-core 2.8 GHz Intel Xeon machine with data on a network disk (uncached I/O read and write speeds of roughly 100 MB s^−1^). However, in extreme cases such as with the urease, which has a total of 20 million reflections, run times were routinely 12 h. Therefore, a faster step for the generation of merging statistics would significantly reduce the generation time and total run time of the program. While we have not employed such a concept in this initial study, we are also looking into the use of a ‘free’ set of reflections similar to *R*_free_ (Brünger, 1992 ▸) or CC_free_ (Karplus & Diederichs, 2012 ▸). Finally, it might also be possible to use the GA approach to optimize the merging of still diffraction frames generated by XFEL data collections. Thus, there are numerous improvements envisioned for this approach based on this proof of concept. However, even in this initial state, the GA is a promising technique for treating SX data and offers a complementary approach to existing methods for treating SX data.

## Figures and Tables

**Table 1:** Crystal and data-collection parameters

Macromolecule	Glucose isomerase	Ultralente insulin	Thermolysin	LUX–DNA	Urease
Space group	*I*222	*H*3	*P*6_1_22	*P*1	*P*6_3_22
Unit-cell parameters (Å, °)	*a* = 93.1, *b* = 99.5, *c* = 102.4	*a* = 82.2, *b* = 82.2, *c* = 33.8	*a* = 92.9, *b* = 92.9, *c* = 129.5	*a* = 32.9, *b* = 70.9, *c* = 67.0, α = 103.9, β = 92.4, γ = 91.0	*a* = *b* = 132.43, *c* = 190.6,
Beamline	ID23-EH2, ESRF	ID23-EH2, ESRF	ID29, ESRF	ID23-EH2, ESRF	P13, PETRA III
Wavelength (Å)	0.8731	0.8731	1.280	0.8731	2.0664
Beam size (H × V or diameter) (µm)	9 × 5	9 × 5	10 × 10	9 × 5	30
Crystal size range (µm)	10 × 10 × 10–30 × 30 × 30	5 × 5 × 5–15 × 15 × 15	20 × 20 × 100	25 × 5 × 5–100 × 5 × 5	20 × 20 × 40–20 × 20 × 70
Photon flux (photons s^−1^)	1.6 × 10^11^	7.0 × 10^10^	4.1 × 10^11^, 8.4 × 10^11^	4.4 × 10^10^	3.4 × 10^11^
Exposure per image (s)	0.1	0.25	0.037	0.1	0.04
No. of images per sub-data set	140	100	100	100	300
Dose per sub-data set (average diffraction-weighted dose) (MGy)	6.0–7.3	5.3–7.5	3.0–6.2	1.67	0.48
Dose per sub-data set (average dose exposed region) (MGy)	1.1–7.8	2.96–10.5	4.2–8.7	0.20–0.63	0.82
Oscillation range (°)	0.1	0.1	0.1	0.1	0.1
Total angular range per sub-data set (°)	14	10	10	10	30

**Table:** Three columns are used for each system, with the first listing data resulting from merging all sub-data sets, the next from the best GA run and the last from the best HCA cluster. Note that for average sub-data-set parameters, not all sub-data sets contained enough reflections to calculate merging statistics [*R*_meas_ and 〈*I*/σ(*I*)〉].

	Glucose isomerase			Ultralente insulin			Thermolysin		
	All	GA	HCA	All	GA	HCA	All	GA	HCA
No. of sub-data sets	30	21	20	53	30	19	206	8	36
HCA CC cutoff	—	—	0.98	—	—	0.97	—	—	0.93
GA population size	—	20	—	—	25	—	—	20	—
GA generations	—	300	—	—	300	—	—	60	—
GA *R* weight	—	0.5	—	—	4	—	—	8	—
GA *I* weight	—	1.5	—	—	4	—	—	1	—
GA CC_1/2_ weight	—	2	—	—	5	—	—		—
GA groups	—	3	—	—	3	—	—	8	—
Data sets in common between GA and HCA	—	19	—	—	15	—	—	0	—
Sub data-set *R*_meas,inner_ †	20.6 (22.9)	9.0 (3.9)	9.8 (3.9)	9.7 (6.5)	8.5 (5.7)	9.4 (5.1)	27.2 (27.8)	5.6 (2.3)	10.2 (3.8)
Sub data-set 〈*I*/σ(*I*)〉_inner_ †	7.6 (4.6)	9.7 (3.7)	9.8 (3.9)	14.8 (8.5)	15.4(8.2)	17.6 (8.9)	7.2 (5.8)	15.4 (4.8)	9.2 (4.5)
Sub data-set completeness† (%)	23.8 (1.4)	24.4 (0.8)	24.4 (0.8)	13.9 (0.7)	13.9 (0.8)	14.0 (0.6)	44.8 (4.4)	46.1 (3.8)	43.9 (3.3)
Resolution range (Å)									
Overall	46.6–1.53	46.6–1.53	46.6–1.53	41.13–1.50	41.13–1.50	41.13–1.50	46.5–1.65	46.5–1.65	46.5–1.65
Outer shell	1.57–1.53	1.57–1.53	1.57–1.53	1.54–1.50	1.54–1.50	1.54–1.50	1.69–1.65	1.69–1.65	1.69–1.65
Total No. of reflections									
Overall	1111281	784525	751377	2019411	114748	72438	8387107	322089	152135
Outer shell	77220	54777	52212	14256	8106	5326	958429	24310	103756
No. of unique reflections									
Overall	71803	71520	72007	13640	13631	13547	40366	39986	40387
Outer shell	5281	5243	5316	1005	1004	1037	2758	2943	2756
Completeness (%)									
Inner shell	99.3	98.9	98.9	98.7	99.3	99.3	99.8	99.3	99.5
Outer shell	100.0	99.8	99.9	100.0	99.9	99.7	100.0	100.0	100.0
Overall	100.0	99.9	99.6	100.0	99.9	99.5	100.0	99.0	100.0
Multiplicity									
Inner shell	14.8	10.4	10.0	16.1	9.2	5.8	192.1	7.3	34.3
Outer shell	14.6	10.4	9.8	14.2	8.1	5.1	205.7	8.3	37.6
Overall	15.5	10.9	10.4	14.8	8.4	5.3	207.7	8.0	37.7
*R* factor (%)									
Inner shell	14.7	9.4	8.0	43.4	6.8	7.0	39.0	8.7	11.8
Outer shell	249.1	153.8	170.0	108.1	85.4	74.5	379.0	171.2	119.7
Overall	33.9	20.4	21.3	34.0	9.1	9.2	91.3	27.4	25.2
*R*_meas_ (%)									
Inner shell	15.2	9.9	8.4	44.8	7.2	7.6	39.2	9.4	12.0
Outer shell	258.1	161.5	179.1	112.1	91.2	82.9	380.7	182.5	121.3
Overall	35.1	21.4	22.3	35.3	9.7	10.2	91.6	29.5	25.5
〈*I*/σ(*I*)〉									
Inner shell	27.9	27.1	26.8	31.9	33.4	23.4	66.2	99.4	27.3
Outer shell	2.5	2.5	2.4	2.6	2.4	1.9	1.4	1.6	4.1
Overall	10.7	10.6	10.3	13.3	12.9	9.7	15.2	17.0	12.9
SigAno									
Inner shell	—	—	—	—	—	—	—	—	—
Outer shell	—	—	—	—	—	—	—	—	—
Overall	—	—	—	—	—	—	—	—	—
CC_1/2_(%)									
Inner shell	97.9	99.5	99.5	99.7	99.8	99.7	97.7	98.2	99.5
Outer shell	68.0	70.8	66.6	77.4	79.1	67.3	69.7	55.3	91.5
Overall	99.2	99.5	99.5	99.3	99.8	99.7	99.1	91.7	99.7

**Table:** 

	LUX–DNA			Urease		
	All	GA	HCA	All	GA	HCA
No. of sub-data sets	204	36	77	127	39	79
HCA CC cutoff	—	—	0.75	—	—	0.8
GA population size	—	25	—	—	20	—
GA generations	—	300	—	—	250	—
GA *R* weight	—	1	—	—	1	—
GA *I* weight	—	2.5	—	—	3	—
GA CC_1/2_ weight	—	1	—	—	2	—
GA groups	—	3	—	—	3	—
Data sets in common between GA and HCA	—	26	—	—	34	—
Sub data-set *R*_meas,inner_ †	‡	‡	‡	15.3 (18.9)	5.2 (3.0)	6.5 (3.4)
Sub data-set 〈*I*/σ(*I*)〉_inner_ †	‡	‡	‡	10.9 (6.7)	16.6 (6.2)	14.1 (5.8)
Sub data-set completeness† (%)	5.4 (1.2)	5.7 (0.9)	5.8 (0.8)	42.9 (4.9)	43.9 (2.3)	43.5 (3.1)
Resolution range (Å)						
Overall	68.86–2.80	68.86–2.80	68.86–2.80	98.38–2.09	98.38–2.09	98.38–2.09
Outer shell	2.87–2.80	2.87–2.80	2.87–2.80	2.14–2.09	2.14–2.09	2.14–2.09
Total No. of reflections						
Overall	292976	70083	118205	20341640	5794906	12815153
Outer shell	16922	4231	7949	749997	182806	608053
No. of unique reflections						
Overall	14499	14224	14024	107644	107603	105577
Outer shell	1039	928	1000	7320	7016	7173
Completeness (%)						
Inner shell	100.0	94.5	96.2	99.9	99.9	100.0
Outer shell	100.1	89.3	99.5	100.0	87.5	100.0
Overall	99.9	98.0	99.4	100.0	99.1	100.0
Multiplicity						
Inner shell	22.6	5.3	9.0	224.3	66.9	146.0
Outer shell	16.3	4.1	7.9	102.4	22.8	84.8
Overall	20.2	4.8	8.4	189.0	53.4	121.4
*R* factor (%)						
Inner shell	71.0	17.0	32.6	93.5	6.7	8.4
Outer shell	187.0	83.2	141.3	157.8	128.8	145.0
Overall	74.5	37.0	50.3	86.0	29.8	32.0
*R*_meas_ (%)						
Inner shell	72.7	18.8	35.1	93.6	6.7	8.4
Outer shell	192.6	93.5	151.2	158.6	131.3	145.8
Overall	76.1	41.0	52.9	86.2	30.1	32.2
〈*I*/σ(*I*)〉						
Inner shell	11.8	16.3	11.3	101.1	121.4	102.9
Outer shell	3.7	5.0	4.8	3.4	2.4	4.2
Overall	7.8	9.9	7.5	24.9	23.3	25.8
SigAno						
Inner shell	—	—	—	3.16	3.79	3.27
Outer shell	—	—	—	0.73	0.76	0.73
Overall	—	—	—	0.99	1.05	1.00
CC_1/2_ (%)						
Inner shell	93.7	95.0	98.1	99.9	99.6	99.9
Outer shell	68.1	59.6	44.9	80.5	66.6	92.8
Overall	94.4	94.3	97.0	99.9	99.8	99.9

† Average values; standard deviations are given in parentheses.

‡ Insufficient reflections in the sub-data sets to obtain statistics.
